# Transbronchial cryobiopsy is safe for patients with echocardiographic evidence of pulmonary hypertension: a blinded prospective cohort study

**DOI:** 10.3389/fmed.2026.1842852

**Published:** 2026-05-07

**Authors:** Hadas Gilboa-Sagy, Ayal Romem, Elad Guber, Gali Epstein Shochet, Andy Eyre, Yoram Neuman, David Shitrit

**Affiliations:** 1 Department of Pulmonology, Meir Medical Center, Kfar Saba, Israel; 2 Faculty of Medicine, Tel Aviv University, Tel Aviv, Israel; 3 Department of Cardiology, Meir Medical Center, Kfar Saba, Israel

**Keywords:** bleeding, cryo-biopsy, echocardiography, pulmonary hypertension, safety

## Abstract

**Introduction:**

Bleeding is the most concerning complication of transbronchial lung cryobiopsy (TBCB). Pulmonary hypertension (PH) is frequently treated as a relative contraindication to bronchoscopic biopsy because of presumed excess bleeding risk, yet TBCB specific data is limited.

**Methods:**

We conducted a prospective blinded cohort study including consecutive adult patients with interstitial lung disease (ILD) referred to TBCB. All participants underwent pre-procedure transthoracic echocardiography (TTE) with estimation of pulmonary artery systolic pressure (EPASP); bronchoscopists were blinded to TTE results. PH was defined as absent, mild (EPASP 37–50 mmHg) or moderate (51–70 mmHg). Severely elevated EPASP (>70 mmHg) was planned to trigger unblinding but did not occur. Primary outcomes were quantitative bleeding volume, measured by net suction volume, and clinically graded bleeding severity. Secondary outcomes included pneumothorax, hypoxemia, and hypotension.

**Results:**

A total of 74 patients were included; 19 patients met any PH definition (EPASP>36 mmHg). There was an insignificant age difference between any-PH and no-PH groups (66.6 ± 13.4 vs. 59.5 ± 13.2 years, *p* = 0.052), a slight male preponderance, and increased hypertension and diabetes rates in the any-PH group. There was no association between PH status and bleeding volume (Mann–Whitney-U, *p* = 0.54), nor across PH severity strata (Kruskal–Wallis, *p* = 0.52). This was further validated by multivariable log-linear regression. One hypoxemia event occurred; Pneumothorax occurred in 6.8%, with no association with PH (*p* = 0.51); no hypotension events were observed.

**Conclusion:**

TBCB is safe among patients with echocardiographic evidence of mild to moderate PH. These findings should be further studied, yet provide positive prospective evidence.

## Introduction

Interstitial lung disease (ILD) accounts for approximately 15% of referrals to general pulmonologists (1). The current approach to diagnosing and managing ILD involves a multidisciplinary discussion among clinicians, radiologists, and pathologists (2). Despite improvements in the specificity of radiological descriptions of ILD, adequate lung biopsies remain essential for accurate diagnosis in many cases (2, 3). Conventional forceps bronchoscopy biopsies are often insufficient to establish a confident diagnosis (4). However, the emergence of transbronchial cryobiopsy (TBCB) provides an effective alternative that is considered safer than surgical lung biopsy (SLB) (5–8). In addition to its higher yield, earlier reports have highlighted a higher complication rate compared to conventional biopsy techniques, mostly in the form of pneumothorax and bleeding (8).

Pulmonary hypertension (PH) is often considered by pulmonologists to be a contraindication for bronchoscopic biopsy due to the possibility of higher risk of bleeding, despite contradictory data in the literature (9–11). Only a few studies have assessed this issue with inconsistent results. One previous publication examined the safety of conventional transbronchial biopsies in COPD patients with documented PH, showing similar bleeding rates between PH and non-PH subjects (12). Regarding TBCB, a recent expert statement noted that PH may increase the risk of bleeding, but no studies have confirmed or refuted this assumption (13).

This prospective double-blinded cohort study in a high-throughput interventional pulmonology unit evaluates the effect of undiagnosed PH, as determined via pre-procedure echocardiography, on the safety of TBCB in a population with suspected ILD.

## Methods

This is a prospective blinded cohort study. The recruitment of all adult patients with suspected ILD referred to TBCB sampling at the interventional pulmonology unit at Meir Medical Center was considered. Exclusion criteria were coagulopathy (platelet count <50,000; international normalized ratio >1.5), inability to withhold anti-aggregation/anti-coagulation medications, excluding aspirin, hemodynamic instability, pregnancy, severe uncorrectable hypoxemia, and patients with known PH. All patients meeting the criteria were offered participation. Patients on NOAC treatment were required to withhold treatment, according to standard practice.

### Echocardiographic assessment of pulmonary hypertension

Following recruitment, all participants underwent transthoracic echocardiography (TTE) in the echocardiography suite at Meir Medical Center, immediately prior to the TBCB procedure. During TTE, the estimated pulmonary artery systolic pressure (EPASP) was calculated as the sum of the tricuspid regurgitation gradient and estimated right atrial pressure, provided there was no increase in pulmonic valve flow velocity.

PH was defined as EPASP >36 mmHg. Patients thus classified with echocardiographic PH were further divided into two additional groups: mild (37–50 mmHg) and moderate (51–70 mmHg), according to recent guidelines (14).

Cardiology staff were blinded as to clinical details of the patients, and in particular, the reasons for and details of the planned pulmonology procedure. Patients and all staff outside of the echocardiography unit were blinded as to the results of the TTE. An exception to the above protocol was made in the case of an estimated EPASP of 70 mmHg or higher, in which case the cardiologist performing the TTE immediately notified the attending interventional pulmonologist to reconsider whether to proceed with the TBCB procedure in view of this finding.

### Transbronchial cryobiopsy

All TBCB procedures were performed by two interventional bronchoscopists, with the patient under general anesthesia or deep sedation with spontaneous breathing. An endotracheal tube was used to secure the airway. TBCB was performed using two flexible bronchoscopes (Olympus TH 190; Olympus, Tokyo, Japan), using standard technique (15). An Erbe (Tübingen, Germany) 1.9 mm (reusable) cryoprobe or 1.7 mm (single use) cryoprobe was advanced through the working channel of the first bronchoscope to the distal lung parenchyma in the target area under fluoroscopic guidance. The probe was withdrawn approximately 1 cm from the point of resistance, and cryofreezing was activated with freeze times of 3 s and extended for up to 8 s in cases where the samples were too small. The bronchoscope and cryoprobe were then withdrawn en-bloc and handed to an assistant. Immediately, the second bronchoscope was advanced into the airway and wedged at the target bronchus to control bleeding. Once adequately thawed, the specimen was removed from the probe and preserved in a formalin solution. All patients underwent a plain chest radiograph immediately following the procedure and were monitored in a recovery unit for an additional two hours before being considered for discharge.

### Hemodynamic monitoring

During and after the procedure, vital signs were closely monitored for procedure-related complications. The following complications were recorded: hypoxemia (defined as SpO2 < 90% or an increased need for supplemental oxygen lasting more than one minute, during or within 20 min of a completed procedure), hypotension (defined as mean arterial pressure <60 mmHg or systolic blood pressure <90 mmHg for more than 5 min or the need for administration of vasopressors), any quantity of bleeding, any pneumothorax, and the need for any unplanned medical assessment, medical intervention, or admission.

### Bleeding measurement

Bleeding during the procedure was assessed both quantitatively and according to clinical effects. Quantitative measurement was based on the volume of fluids suctioned through the bronchoscope after subtracting instilled fluids. Clinical effects of bleeding were graded from 0 to 4 on a standard scale: grade 0, traces not requiring suction; grade 1, mild bleeding requiring suction only; grade 2, moderate bleeding requiring wedging of bronchoscope in the biopsied segment for at least 2 min; grade 3, severe bleeding requiring topical epinephrine, aminocaproic acid, cold saline, selective main stem intubation, or bronchial blocker inflation; and grade 4, life-threatening bleeding requiring hemodynamic support, transfusion of blood products, and hospitalization with or without ICU admission, as previously described (16, 17).

### Data collection

Clinical data related to the procedure and its complications was recorded in the hospital electronic patient records (EPR) system immediately after each procedure, according to standard practice. The echocardiography report was also recorded on the EPR system but remained embargoed until after the TBCB was completed.

Data required for the study were subsequently extracted from the EPR system at a later date. These data included: patient demographics (age, BMI, sex, smoking status), medical history (ASA score, comorbidities: congestive heart failure, chronic renal failure, COPD, diabetes mellitus, hypertension, ischemic heart disease, and medication usage: aspirin, clopidogrel, vitamin-K antagonist, direct oral anticoagulants (DOAC), low molecular weight heparin), laboratory values (hemoglobin, platelet count, PT, PTT, creatinine, urea), the EPASP as measured by pre-procedure TTE, anatomic details of the TBCB (the target lobe and number of biopsies taken), the resulting pathological diagnosis, and any complications (bleeding grade, bleeding volume, pneumothorax, and periprocedural hypoxia or hypotension).

Pathological diagnosis was further categorized as fibrotic or non-fibrotic. Fibrotic diseases were considered as: any UIP, any silicosis, and any other primary pathology (such as NSIP or HP) where fibrosis was also noted to be present.

### Statistical analysis

The data were analyzed using R version 4.5.1 and RStudio version 2025.05.1. Summary statistics for most continuous variables are reported as mean ± sample standard deviation, except for bleeding volume which has a skewed distribution as is reported as median volume with IQR. Binary variables are reported as frequencies along with 95% CI. Between group comparisons for means of continuous variables were tested using Welch’s *t*-test, and between group comparisons for binary variables were tested using Fisher’s exact test. Uniformity of distribution within a given categorical variable was tested using a χ^2^ goodness-of-fit against the uniform null hypothesis. Differences in the distribution of ordinal or continuous variables between groups were tested with the nonparametric Mann–Whitney U-test. Nonparametric testing of continuous variables between three groups used the Kruskal-Wallis rank sum test. Nonparametric testing of associations between two continuous variables used Spearman’s rank correlation coefficient. Where appropriate, false discovery rate was controlled for multiplicity using the Benjamini-Hochberg (BH) procedure. Effect size for bleeding volume used the Hodges-Lehmann estimate.

A single outlying datum with an extreme bleed volume of 500 mL was excluded from the final analysis, based on a robust Z score of 14.2. The bleeding was adjudged clinically unrelated to cardiopulmonary status, which would be classified as mild PH according to EPASP.

Sensitivity analysis for the inclusion of this patient showed no change in estimates of the difference in bleeding volume between patients with and without PH and did not alter the significance of analyses or conclusions (Supplementary material).

## Results

75 patients were prospectively included in the study. After exclusion of one single outlier with an extremely high bleeding volume (see statistical analysis section above), 74 patients entered the final analysis. Of these, 19 patients had EPASP >36 mmHg. The mean EPASP for the study population was 36 ± 11 mmHg, and no patients were excluded due to extremely elevated EPASP (>70 mmHg). Mean EPASP was 48.3 ± 9.9 mmHg in the any-PH group versus 29.7 ± 3.9 mmHg in the no-PH group (*p* < 0.001).

Demographic and clinical characteristics of the cohort are presented in Table 1. There was a marginally statistically significant difference in age between any-PH and no-PH groups (66.6 ± 13.4 vs. 59.5 ± 13.2 years, *p* = 0.052). There was a slight male preponderance in the cohort overall, with no statistically significant difference between groups (Table 1, *p* = 0.41). Other demographic, clinical, and comorbidity variables were comparable, except for higher rates of NOAC use (15.8% [95% CI 3.0–40.0] vs. 1.9% [95% CI 0.0–10.0], *p* = 0.05) and aspirin use (58.0% [95% CI 34.0–80.0] vs. 20.0% [95% CI 10.0–33.0], *p* = 0.03) in the PH group. Hypertension, and diabetes mellitus were also more frequent in the PH group (Table 1). Clopidogrel use was minimal in both groups. No significant difference in anesthetic risk as measured by ASA grade was observed (Mann–Whitney U, *p* = 0.42).

**Table 1:** Patient characteristics.

Parameter	No PH *n* = 55	PH *n* = 19	*p*-value
Age (years)	59.47 ± 13.22	66.63 ± 13.37	0.052
Male	61.8% (0.477–0.746)	73.7% (0.488–0.909)	0.414
BMI	28.45 ± 5.088	27.64 ± 5.044	0.555
Smoker	60% (0.459–0.73)	73.7% (0.488–0.909)	0.408
ASA grade 3	32.7%	36.8%	0.42
ASA grade 2	58.2%	31.6%	
Comorbidities			
CHF	3.6% (0.004–0.125)	15.8% (0.034–0.396)	0.103
CRF	10.9% (0.041–0.222)	0% (0–0.176)	0.328
COPD	12.7% (0.053–0.245)	26.3% (0.091–0.512)	0.276
Diabetes mellitus	25.5% (0.147–0.39)	52.6% (0.289–0.756)	0.046
Hypertension	32.7% (0.207–0.467)	68.4% (0.434–0.874)	0.014
IHD	20% (0.104–0.33)	36.8% (0.163–0.616)	0.213
Lab tests			
Hemoglobin	13.856 ± 1.585	14.195 ± 1.667	0.446
INR	0.977 ± 0.11	1.031 ± 0.109	0.084
PTT	26.253 ± 4.335	26.431 ± 2.526	0.849
PLT	243.509 ± 80.347	237.579 ± 88.111	0.798
Creatinine	0.991 ± 0.679	0.951 ± 0.292	0.728
Urea	37.957 ± 24.631	42.276 ± 22.11	0.491
Relevant medication			
Aspirin	20% (0.104–0.33)	57.9% (0.335–0.797)	0.003
Clopidogrel	5.5% (0.011–0.151)	0% (0–0.176)	0.565
NOAC*	1.8% (0–0.097)	15.8% (0.034–0.396)	0.05

ASA, American society of anaesthesiologists; CHF, Chronic Heart Failure; COPD, Chronic Obstructive Pulmonary Disease; CRF, Chronic Renal Failure; IHD, ischemic heart disease; INR, International Normalized Ratio; NOAC, Novel Oral Anticoagulants; *Taken prior to the procedure, PH, pulmonary hypertension; PTT, Partial Thromboplastin Time; PLT, platelet count.

Most patients underwent biopsy of the right lung (93.0% [95% CI 85.0–98.0]), with far fewer left-lung biopsies (5.0% [95% CI 1.5–13.0]) and bilateral procedures (1.0% [95% CI 0.0–7.0]), and this difference is statistically significant (χ^2^ test for uniformity, *p* < 0.001). The majority of patients had biopsies taken from all ipsilateral lobes (73.0% [95% CI 61.0–83.0]). There was no difference in anatomical distribution of target sites between PH and non-PH groups (Fisher’s exact test, *p* = 0.30). The mean number of biopsy samples per patient was 3.3 ± 0.7, with no difference in the mean number of biopsies between the PH and non-PH groups (Mann–Whitney U, *p* = 0.99).

The most frequent histological pattern was NSIP (27.0% [95% CI 17.4–38.6]), followed by HP/BIP (18.9% [95% CI 10.7–29.7]) and UIP (17.6% [95% CI 9.7–28.2]). Histological sarcoidosis accounted for 4.1% (95% CI 0.8–11.4). Overall, 35.1% (95% CI 24.4–47.1) of patients had biopsy evidence of a fibrotic ILD. Presence of any fibrotic pattern was associated with a higher likelihood of PH (42.3% [95% CI 23.4–63.1] of the fibrotic cohort; OR 3.6 [95% CI 1.1–12.6], *p* = 0.025). Having histological UIP specifically was associated with a higher prevalence of PH that did not reach statistical significance (46.2% [95% CI 19.2–74.9]; OR 3.1 [95% CI 0.7–13.1], *p* = 0.08). No other pathological diagnosis, fibrotic or otherwise, was significantly associated with PH.

**Figure 1:**
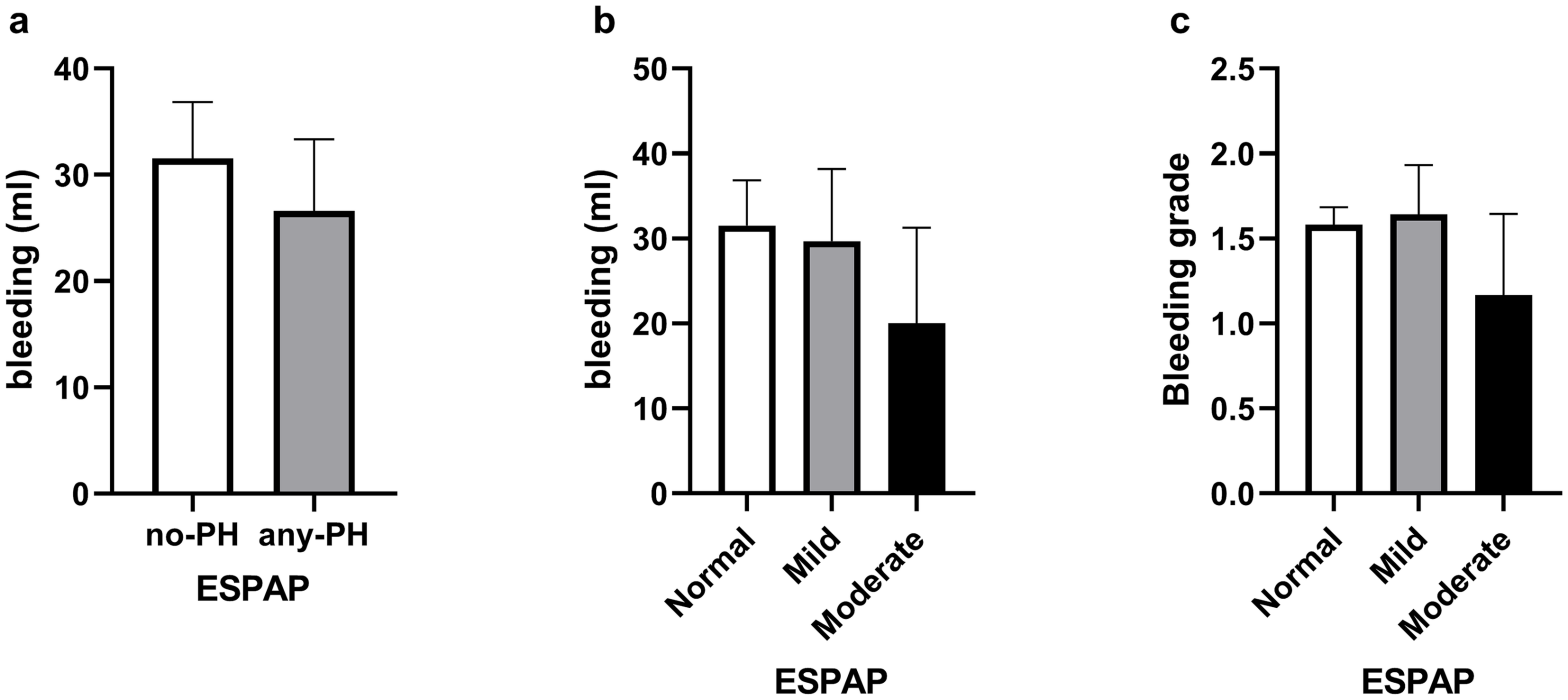
Histograms of bleeding volume (mL) and bleeding severity. **(a)** Average bleeding volume (mL) for the entire cohort by PH status; **(b)** average bleeding volume (mL) for the entire cohort by PH severity and **(c)** bleeding severity category for the entire cohort by PH severity. Data is presented with average ± SEM. ESPAP, estimation of pulmonary artery systolic pressure; PH categories included Normal PH (<36 mmHg), mild (37–50 mmHg) and moderate (51–70 mmHg).

No association was observed between bleeding volume and presence of PH. The Hodges–Lehmann estimate for the difference in bleeding volume was −2 mL for PH versus no PH (95% CI, −12 to 9 mL, Mann–Whitney U, *p* = 0.54, Figure 1a), indicating little meaningful difference in bleeding volume between groups. Sensitivity analyses showed that this finding was unchanged when varying the EPASP threshold used to define PH (Supplementary Figures S1, S2) and when stratifying patients according to baseline anticoagulant and antiplatelet therapy, with no association between PH and bleeding volume in either subgroup (Supplementary material).

No association was found between the quantity of bleeding and PH when classified as either absent, mild or moderate (Kruskal-Wallis, *p* = 0.52, Figure 1b). Multiple univariable analyses across demographic, clinical, and laboratory variables failed to find any significant association with bleeding volume after correction for false discovery rate. Moreover, no association was found between bleeding grade and the presence or absence of PH (Mann–Whitney U, *p* = 0.45), nor was any association found between bleeding grade and PH status stratified by severity (Kruskal-Wallis, *p* = 0.54, Figure 1c). Distributions of bleeding volume and grades, stratified by PH severity, are presented in Supplementary Figures S3, S4.

Multivariable log-linear regression confirmed the lack of association between measured EPASP and bleeding volume. Of the included variables, only platelet count (bleeding volume ratio 0.43 per 100-unit increase; 95% CI 0.21–0.87; *p* = 0.02) and PTT (bleeding volume ratio 4.7 per 10-s increase; 95% CI 1.15–19.5; *p* = 0.03) were independently associated with bleeding volume (Supplementary Table 1). NOAC use showed a borderline negative association (bleeding volume ratio 0.11; 95% CI 0.01–1.1; *p* = 0.06), likely reflecting procedural conservatism in patients presumed to be at higher bleeding risk. Age, sex, BMI, hemoglobin, creatinine, ASA grade, number of biopsies, EPASP, and aspirin use were included in the model but were not independently associated with bleeding volume.

The overall pneumothorax rate was 6.8% (95% CI 2.2–15.1). No association was found between pneumothorax and PH status (2/19 vs. 3/55 cases of pneumothorax, for PH vs. no PH, respectively, Fisher’s exact test, *p* = 0.51), nor any other predictor, based on multiple univariable testing. A single hypoxic event occurred during the study, in a patient with mild PH (event rate 1.4%; 95% CI 0.0–7.3). No episodes of hypotension were observed (event rate 0.0%; 95% CI 0.0–4.9). Multiple univariable analyses across demographic, clinical, and laboratory variables failed to find any significant association with bleeding volume after correction for false-discovery rate (Supplementary Table 2).

## Discussion

In this prospective blinded cohort study of 74 patients, we examined complication rates for TBCB for ILD with respect to preprocedural echocardiographic estimation of pulmonary artery systolic pressure (EPASP). We found no association between EPASP and bleeding volume during the procedure. Of the clinical variables considered, the only ones correlated with an increased bleeding volume were a lower platelet count and a higher PTT. Neither aspirin use nor NOAC use as regular medications prior to the procedure were associated with increased bleeding risk. No association was found between bleeding grade on an ordinal scale and EPASP, nor any other clinical predictor. No association was found between the risk of pneumothorax and any of the clinical variables considered.

In recent years, TBCB has become the preferred diagnostic tool for patients with ILD who require histopathology for conclusive diagnosis, as reflected in the official clinical practice guidelines by ATS/ERS/JRS/ALAT (18, 19). For most indications TBCB has supplanted SLB, which remains the gold standard in terms of pathological yield, but is associated with significant comorbidity and whose use is limited in patients with poor cardiovascular or pulmonary status (20, 21). Studies have shown good agreement in both diagnostic yield and accuracy between TBCB and SLB, as recently reviewed in (8). For instance, the COLDICE study demonstrated a 76.9% agreement between TBCB and SLB in the diagnosis as determined by multidisciplinary discussion, with a similar degree of contribution (74% in TBCB vs. 77% in SLB). The study reported a bleeding incidence of 22%, while the incidence of pneumothorax could not be ascertained (22, 23).

Bleeding is the most concerning complication of TBCB. Published meta-analyses report a wide range of substantial bleeding, ranging between 0.3–26% of cases with unknown certainty due to the variability of bleeding definition and the different methods utilized for bleeding control.

PH is considered a relative contraindication for endoscopic biopsy due to the presumed increased risk of bleeding, despite the lack of evidence to substantiate this assumption. Proposed explanations include submucosal bronchial vein and plexus dilatation resulting in an increased tendency for bleeding. Other safety concerns in patients with PH include right ventricular dysfunction, which may cause ischemia, arrhythmia, and hypotension, and disrupted physiological VQ matching, resulting in hypoxia (10, 11, 24, 25).

Bondue et al. (26) was the first prospective study to assess the safety of TBCB that included patients with echocardiographic PH. The study included 98 patients divided into high and low-risk groups. The high-risk group included patients with advanced age, obesity, reduced ejection fraction, and impaired lung function, in addition to EPASP ≥ 45 mmHg on echocardiography. Although no significant difference was found in complication rates between the groups, only four out of the high-risk cohort had raised EPASP, limited the applicability of the conclusions to patients with PH (26).

The current study demonstrated that TBCB is safe among patients with mild to moderate echocardiographic PH. This is despite the higher rate of undisrupted aspirin use as well as temporarily withheld chronic NOAC use among the PH group, which in any case were not found to be associated with a higher volume of bleeding. The study also showed low overall rates of other complications, including pneumothorax and hypoxemia and hypotension, and no significant association with echocardiographic PH. These results correspond to previous findings, which showed that continuation of aspirin is safe for TBCB procedures (27).

Our results are consistent with previous studies assessing the safety of bronchoscopy with transbronchial biopsy (TBB), which show that mild PH is not associated with an increased risk of bleeding. Neuman et al. (12) demonstrated that TBB is safe among patients with mild PH and COPD, with no evidence for greater risk of bleeding. Likewise, the meta-analysis of Ali et al. (28) showed no association between bleeding risk and PH during TBB for a variety of indications.

A key strength of the study is the blinded, prospective design, which utilized preprocedural echocardiography, with bronchoscopists being blind to echocardiography results, and cardiologists to patient history. Another strength is the study population which was a broad and representative sample of ILD patients in secondary care.

Our study has several limitations. Firstly, pulmonary arterial pressure was estimated by transthoracic echocardiography (TTE) and not by right heart catheterization, which is considered the gold standard for diagnosing PH. Nonetheless, TTE derived EPASP remains a practical non-invasive alternative for assessing pulmonary pressure that is widely used in screening patients for PH (29). Secondly, measurement of bleeding quantity by suction volume disregards blood retained in the airways and parenchyma, leading to underestimation. Despite this, this method of estimation has the advantage of consistency, unlike alternative qualitative bleeding assessment grades that are subjective and operator dependent. Thirdly, the limited sample size means that the study was only sensitive to detect moderate or large differences in bleeding volume, as reflected by the confidence interval width of approximately 20 mL in estimates for this difference based on the actual results.

Fourthly, all procedures were performed at a single high-volume center by two experienced bronchoscopists. Although contributing to consistency, results might not be generalized to lower-volume centers or less experienced operators.

In conclusion, our study found no association between EPASP as estimated from pre-procedural TTE and complication rates of TBCB, including procedural bleeding volumes. Known or suspected mild to moderate echocardiographic PH appears not to contraindicate TBCB in a general ILD cohort. These findings should be further validated in a larger ILD cohort.

## Data Availability

The raw data supporting the conclusions of this article will be made available by the authors, without undue reservation.
