# Supplementary material for: Transbronchial cryobiopsy is safe for patients with echocardiographic evidence of pulmonary hypertension: a blinded prospective cohort study
Source: Front Med (Lausanne). 2026 May 7;13:1842852. doi: 10.3389/fmed.2026.1842852 (PMC13190548; doi:10.3389/fmed.2026.1842852)
Supplement: Supplementary file 1 [file Data_Sheet_1.docx]

Transbronchial cryobiopsy is safe for patients with echocardiographic evidence of pulmonary hypertension: a blinded prospective cohort study

Supplementary Material

# The effect on inclusion of the single large volume bleed data point on the primary statistical analysis: the difference in bleeding volume between “No PH” and “Any PH” groups.

A single datum with a very large bleed volume was excluded from the primary analysis as a statistical outlier, based on a robust Z score of 14.2. A sensitivity analysis for the exclusion of this point was conducted on the difference in bleeding volume between “any PH” and “no PH” groups. Excluding this data point, the main analysis showed a Hodges–Lehmann estimate for the difference in bleeding volume of -2ml for “any PH” vs “no PH” (CI -12—9ml, Wilcoxon rank sum p=0.5432) which was not statistically significant. Including this data point, the Hodges–Lehmann estimate for the difference in bleeding volume was 0ml (CI -10—15ml, Wilcoxon rank sum p=0.7962). Therefore the inclusion of this outlying datum made essentially no difference to estimates of the difference in bleeding volume between the two groups, which is both cases was not statistically significant.

# The effect of EPASP threshold for the pulmonary hypertension (PH) group on the effect and statistical significance of the analysis of the effect of pulmonary hypertension on bleeding volume

A sensitivity analysis was conducted to determine the effect of shifting the EPASP threshold for defining the “any PH” group of the main statistical analysis of the study, namely, the effect of pulmonary hypertension on the bleeding volume measured during cryobiopsy. Figure 1 shows that at no reasonable EPASP cutoff (in the range 30-45mmHg) is there any statistically significant difference in a Wilcoxon test for bleeding volume for the “any PH” group vs the “no PH” group. Similarly, Figure 2 shows no clear effect on median bleed volume for the two groups as the EPASP cutoff changes within this range.


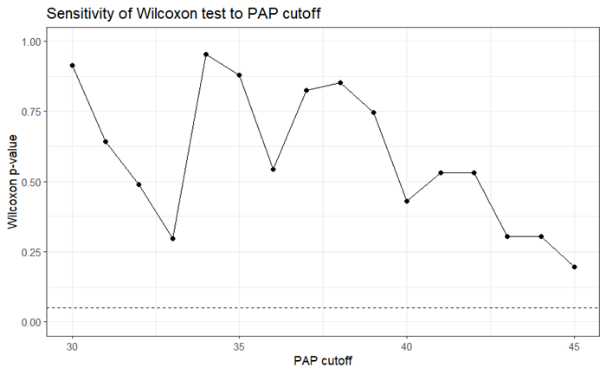


# *Figure 1. The Wilcoxon p-value for the difference in bleeding volume between "any PH" and "no PH" groups, as a function of the EPASP threshold used to define the "any PH" group. As can be seen, at no reasonable EPASP threshold do we see any significant difference between the bleeding volumes of the two groups.*


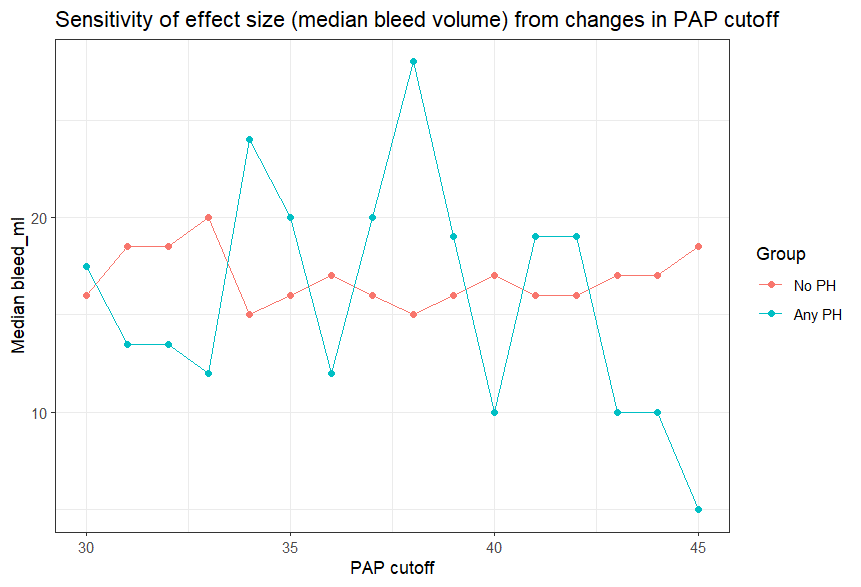


*Figure 2. The changes in the median bleed volume for "any PH" and "no PH" groups, as a function of the EPASP threshold used to define the "any PH" group. There is no clear effect on median bleed volume from change in the EPASP threshold.*

# Univariable analysis of bleeding volume for patients with and without PH, in the subgroups of patients with and without background anticoagulant and antiaggregant use

For anticoagulated patients, the cohort size is n=28 (13 PH, 15 non-PH), and univariable analysis gives a Hodges-Lehrmann estimate for difference in bleeding of -2ml (PH vs non-PH, CI -15—30, Wilcoxon p=0.73). For nonanticoagulated patients, the cohort size is n=46 (6 PH, 40 non-PH) with a Hodges-Lehrmann estimate for bleeding volume difference of -2ml (PH vs non-PH, CI -20—27, Wilcoxon p=0.78). Separating the patients into subgroups based on background anticoagulant and antiaggregant therapy therefore neither changes the effect size (which is identical in the subgroups) nor does it add any new significant association.

**Supplementary Table 1**: Model coefficients for a log-linear multivariable regression of bleeding volume (ml) according to a set of selected demographic and clinical variables. Included variables comprised a set of core variables expected to have clinical effect, in addition to any variables that tested p<0.25 on univariable analysis.

| **Variable** | **p value** | **Ratio** | **95% CI for ratio** |
| --- | --- | --- | --- |
| Intercept | 0.883 | 0.530 | 0.000-3212.099 |
| Age, per year | 0.812 | 1.005 | 0.966-1.045 |
| Male sex | 0.958 | 1.035 | 0.280-3.826 |
| Number of biopsies | 0.181 | 0.647 | 0.338-1.236 |
| Hemoglobin (g/dL) | 0.300 | 1.208 | 0.839-1.739 |
| Creatinine (mg/dL) | 0.710 | 0.673 | 0.079-5.726 |
| Aspirin | 0.258 | 0.498 | 0.146-1.703 |
| NOAC* | 0.060 | 0.107 | 0.010-1.103 |
| EPSAP (severity) | 0.398 | 0.979 | 0.930-1.030 |
| BMI | 0.304 | 1.059 | 0.947-1.183 |
| ASA | 0.507 | 0.763 | 0.336-1.732 |
| Platelets, per 100 units (10^9/L) | 0.020 | 0.425 | 0.208-0.869 |
| PTT, per 10 seconds. | 0.032 | 4.735 | 1.150-19.489 |

American society of anaesthesiologists (ASA), Chronic Renal Failure (CRF), estimation of pulmonary artery systolic pressure (EPSAP), ischemic heart disease (IHD), International Normalized Ratio (INR), Novel Oral Anticoagulants (NOAC) * taken prior to the procedure, Partial Thromboplastin Time (PTT)

**Supplementary Table 2**: Univariable analyses testing for association between demographic and clinic variables, and bleeding volume. Continuous and ordinal variables are tested using Spearman’s test, while binomial variables are tested using the Wilcoxon signed rank test. After correcting for false discovery rate using the Benjamini & Hochberg method, no significant association was found between bleeding volume and any of the variables on test.

| **Variable** | **n** | **Spearman’s rho** | **Median bleed vol (ml)  without risk** | **Median bleed vol (ml)  with risk** | **FDR-adjusted p** |
| --- | --- | --- | --- | --- | --- |
| Demographics | | | | | |
| Age | 74 | 0.0182 |  |  | 0.967 |
| Male sex | 74 |  | 20.5 | 13.5 | 0.967 |
| BMI | 74 | 0.308 |  |  | 0.165 |
| Comorbidities | | | | | |
| Smoker | 74 |  | 15 | 20 | 0.967 |
| Hypertension | 74 |  | 17 | 15 | 0.967 |
| CHF | 74 |  | 20 | 5 | 0.967 |
| IHD | 74 |  | 14.5 | 22.5 | 0.967 |
| COPD | 74 |  | 18.5 | 13.5 | 0.967 |
| CRF | 74 |  | 18.5 | 8.5 | 0.967 |
| Diabetes Mellitus | 74 |  | 18.5 | 13.5 | 0.967 |
| Medication | | | | | |
| Aspirin | 74 |  | 18.5 | 13.5 | 0.967 |
| Clopidogrel | 74 |  | 17 | 15 | 0.967 |
| NOAC* | 74 |  | 16 | 21 | 0.967 |
| Study or procedure related | | | | | |
| ASA grade | 74 | 0.149 |  |  | 0.752 |
| Number of biopsies | 74 | -0.175 |  |  | 0.747 |
| EPASP | 56 | -0.0162 |  |  | 0.967 |
| Laboratory | | | | | |
| Hemoglobin | 74 | 0.178 |  |  | 0.747 |
| Platelets, per 100 units (10^9/L) | 74 | -0.152 |  |  | 0.752 |
| INR | 72 | -0.00686 |  |  | 0.967 |
| PTT | 64 | 0.243 |  |  | 0.581 |
| Urea | 72 | -0.110 |  |  | 0.967 |
| Creatinine | 74 | -0.0130 |  |  | 0.967 |

Chronic Heart Failure (CHF), Chronic Obstructive Pulmonary Disease (COPD), Chronic Renal Failure (CRF), estimation of pulmonary artery systolic pressure (EPSAP), ischemic heart disease (IHD), International Normalized Ratio (INR), Novel Oral Anticoagulants (NOAC) * taken prior to the procedure, pulmonary hypertension (PH), Partial Thromboplastin Time (PTT)


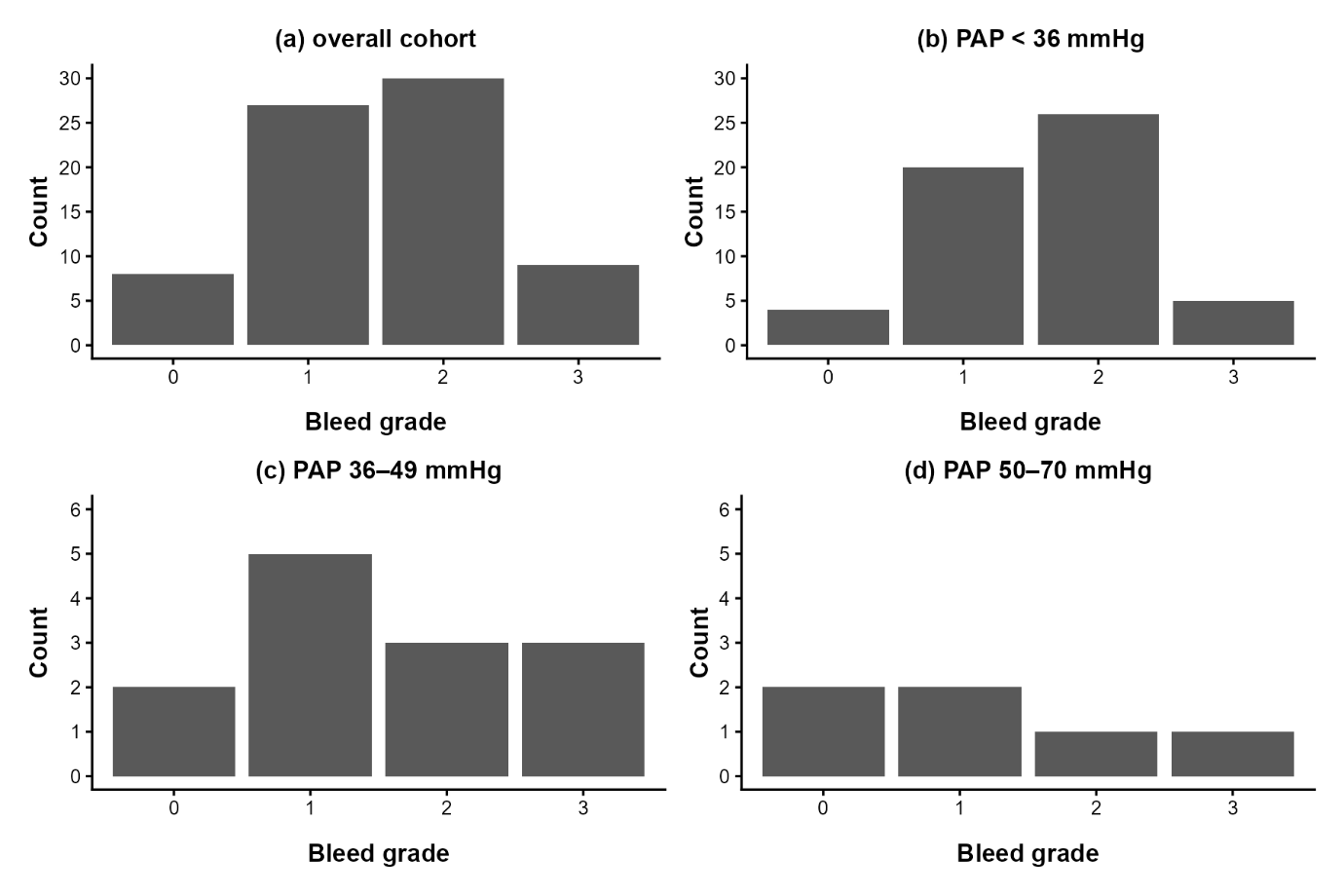


# *Figure 3. Histogram of bleeding grade distribution for the entire cohort (a), as well as for ESPAP severity stratification (b-d). These show that the majority of patients had minor/ moderate bleeding, with similar distribution across ESPAP severity grades.*


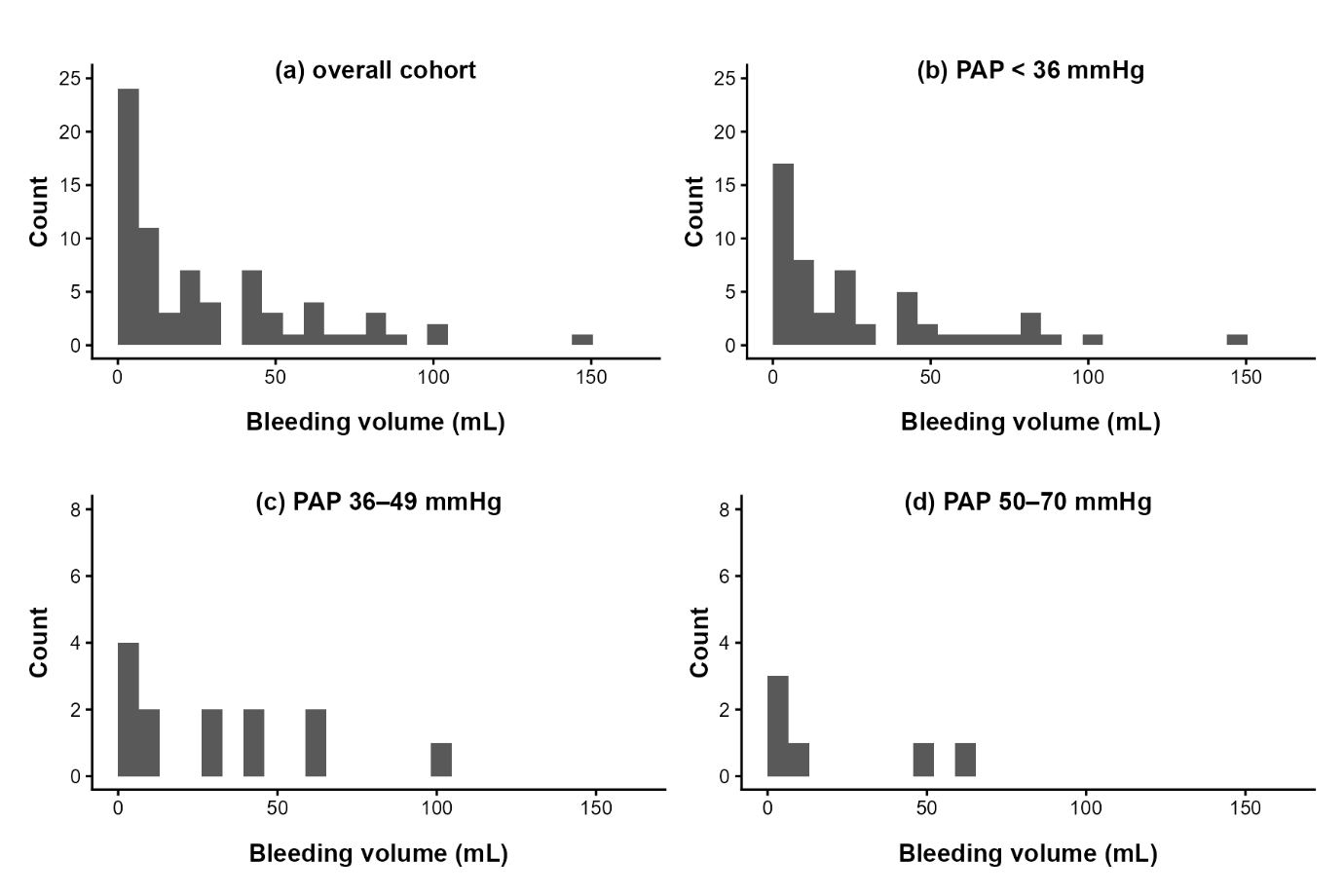


# *Figure 4. Histogram of bleeding volume (ml) distribution for the entire cohort (a), as well as for ESPAP severity stratification (b-d). These show that the majority of patients had minor/ moderate bleeding, with similar distribution across ESPAP severity grades.*
